# HARVESTING SURAL FLAP WITH COVERED PEDICLE

**DOI:** 10.1590/1413-785220233101e257850

**Published:** 2023-04-17

**Authors:** Álvaro Baik Cho, Carlos Henrique Vieira Ferreira, Priscilla Goes Medea de Mendonça, Luiz Sorrenti, Leandro Yoshinobu Kiyohara

**Affiliations:** 1 Universidade de São Paulo, Instituto de Ortopedia e Traumatologia, Department of Hand Surgery and Microsurgery (IOT-FMUSP), São Paulo, SP, Brasil.; 2 Faculdade de Medicina do ABC, Department of Hand Surgery and Microsurgery, Santo Ándre, SP, Brazil.

**Keywords:** Tissue Transplantation, Surgical Flaps, Free Tissue Flaps, Transplante de Tecidos, Retalhos Cirúrgicos, Retalhos de Tecido Biológico

## Abstract

**Objectives:**

The aim was to evaluate the viability and the outcomes of the sural flap performed with the pedicle covered by a strip of skin.

**Methods:**

A prospective cohort of 20 consecutive cases were evaluated in terms of flap viability, complication rate, and the amount of skin graft required. The location of the defects was the middle third of the tibia in 3 cases, the ankle and hindfoot in 15 cases, the middle foot in 1 case, and the forefoot in 1 case. The flap design was the same as described by Masquelet. The only modification included a strip of skin over the entire length of the pedicle. The intermediary skin between the donor site and the defect was incised and the skin was undermined to accommodate the pedicle without compression.

**Results:**

All cases had a satisfactory evolution, with adequate healing and without flap loss. Both the donor site and the pedicle were primarily closed in all cases. In one patient, the flap developed a limited area of superficial epidermolysis that healed spontaneously.

**Conclusion:**

the modified sural flap with a covered pedicle is feasible and reliable with a lower rate of complications when compared with the conventional sural flap. ***Level of Evidence IV, Cohort Studies***.

## RESUMO

**Objetivo:**

O objetivo foi avaliar a viabilidade e os resultados do retalho sural realizado com o pedículo coberto com uma faixa de pele.

**Material e Métodos:**

Pelo estudo de coorte prospectivo, foram avaliados 20 casos consecutivos considerando a viabilidade do retalho, a taxa de complicações e a quantidade de enxerto de pele necessária. A localização dos defeitos foi no terço médio da tíbia em 3 casos, tornozelo e retropé em 15 casos, pé médio em 1 caso e antepé em 1 caso. O desenho do retalho foi o mesmo que o descrito por Masquelet. A única modificação foi a inclusão de uma tira de pele em todo o comprimento do pedículo. A pele intermediária entre o local doador e o defeito foi incisada e a pele foi descolada para acomodar o pedículo sem compressão.

**Resultados:**

Todos os casos tiveram uma evolução satisfatória, com cicatrização adequada e sem perda dos retalhos. Tanto o local doador quanto o pedículo foram primariamente fechados em todos os casos. Em um paciente, o retalho desenvolveu uma área limitada de epidermólise superficial que cicatrizou espontaneamente.

**Conclusão:**

O retalho sural modificado com pedículo coberto é viável e confiável com uma menor taxa de complicações quando comparado ao retalho sural convencional. ***Nível de Evidência IV, Estudos de Coorte***.

## INTRODUCTION

The traditional sural flap as described by Masquelet^1^ is based on the suprafascial course of the sural nerve and includes the lesser saphenous vein for venous return. The pedicle is dissected down to the distal leg until the classic pivot point, about 5 cm above the lateral malleolus^1,2^. It can suffer torsion or compression during transposion of the flap to the recipient area^1-5^. To minimize the risk of pedicle compression, the flap should not be transposed through a subcutaneous tunnel because the skin surrounding the defect might act as a constricting sling due to fibrosis and local edema, jeopardising the venous return. This is often reported as a major cause of flap congestion, resulting in partial or complete flap loss^3^.

The aim of this study is to evaluate the viability and the outcomes of the sural flap performed with a technical modification, in which the pedicle is covered by a strip of skin, avoiding tunnelization of the pedicle.

## PATIENT AND METHODS

Between march 2018 and September 2020, 20 consecutive cases of reverse sural flaps were performed in 20 patients (Table 1). Seventeen were male and three was female. The cause of the soft tissue defect was post-traumatic crushing injuries in 15 cases. The average time from the trauma and the soft-tissue coverage with the sural flap was 1 week. In the other five cases, one was performed for tumor lesion, one was performed for chronic osteomyelitis and the other two for wound dehiscence, one after achilles tendon repair and one after calcaneus osteosynthesis. The location of the defects was the middle third of the tibia in 3 cases, ankle and hindfoot in 15 cases, middle foot in 1 case and forefoot in 1 case. All flaps were performed by three different surgeons in the same University Hospital. The mean age of the patients was 33,1, ranging from 8 to 63 years old. The minimum follow-up required for inclusion in this study was 1 month.

**Table 1:** Patients demographics. Wound location and ethology, flap's and pedicle's length and width and complications.

Patient	Age (year)	Gender	Location of the defect	Etiology	Flap size (length x width) cm	Skin strip size (width x length) cm	Outcomes (Months)	Complications
1	25	Male	Medial Malleolus	Tumor	13 × 5	1,5 × 13	30	None
2	32	Male	Distal Tiibia	Traumatic (motorcycle Accident)	10,0 × 4,0	1,5 × 8	28	None
3	30	Female	Medial Malleolus	Traumatic (Car Accident)	6,0 × 4,5	3 × 10,5	28	None
4	63	Female	AquillesTendon	Post-Op	6,0 × 3,0	1,5 × 7,5	26	None
5	35	Male	Distal Tiibia	Traumatic (motorcycle Accident)	13,0 × 3,5	1 × 8	24	None
6	42	Male	Distal Tibia	Traumatic (motorcycle Accident)	12,0 × 3,0	1,5 × 8	24	None
7	36	Male	Tibial Shaft	Traumatic (motorcycle Accident)	7,5 × 4,3	2,5x 13,0	24	None
8	41	Male	MindFoot	Traumatic (motorcycle Accident)	11,0 × 6,0	2,5 × 14	22	None
9	18	Male	Tibial Shaft	Osteomyelitis chronic	13,5 × 4,5	1,5 × 13	18	None
10	32	Male	Medial Malleolus	Traumatic (motorcycle Accident)	14,0 × 6,5	1,5 × 7	13	None
11	35	Male	Lateral Malleolus	Traumatic (motorcycle Accident)	10 × 5	2 × 14	12	Pedicle Epidermolysis
12	25	Male	ForeFoot	Traumatic (motorcycle Accident)	13,0 × 5,0	1 × 10	11	None
13	30	Male	HindFoot	Chopart Traumatic Amputation	14,5 × 6,5	1,5 × 13,5	11	None
14	60	Male	HindFoot	Post-Op Calcaneous Fracture	4 × 3,0	1,0 × 6,0	7	None
15	8	Male	Tibial Shaft	Traumatic (Running Over)	7,0 × 4,5	1,5 × 5,0	6	None
16	55	Female	AquillesTendon	Post-OP	5,0 × 3,0	2,0 × 5,0	6	None
17	32	Male	Medial Malleolus	Traumatic (motorcycle Accident)	9,0 × 4,0	1,5 × 5,0	5	None
18	25	Male	Medial Malleolus	Traumatic (Running Over)	15,0 × 9,0	2,0 × 10,0	2	None
19	30	Male	Lateral Malleolus	Traumatic (motorcycle Accident)	14,0 × 6,0	2,0 × 9,0	2	None
20	8	Male	Lateral Malleolus	Traumatic (Running Over)	7,0 × 4,5	1,5 × 5,0	1	None

### Surgical Technique

**Figure 1:**
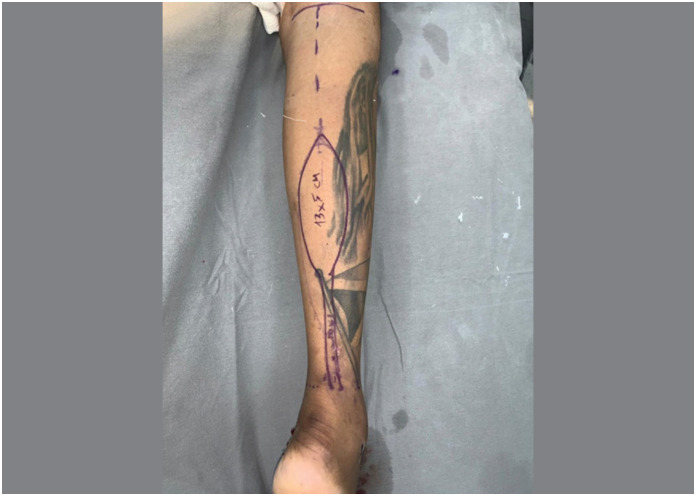
Flap's design. Longitudinal strip of skin in continuity with the flap itself resembling a squash racket.

**Figure 2:**
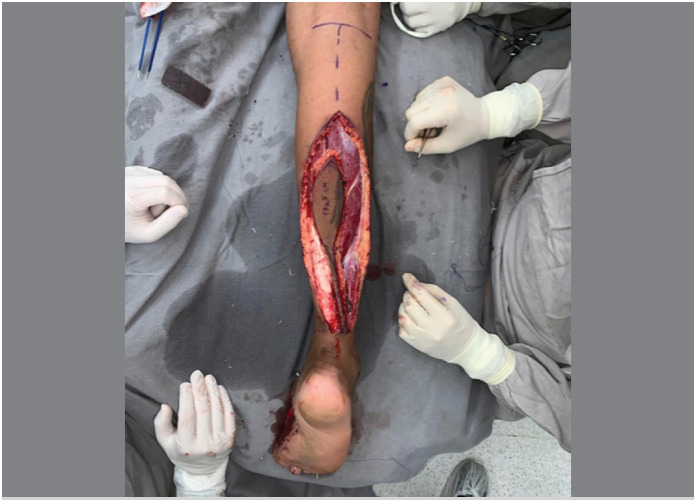
The strip of skin was planned to cover both the sural nerve and the lesser saphenous vein.

**Figure 3:**
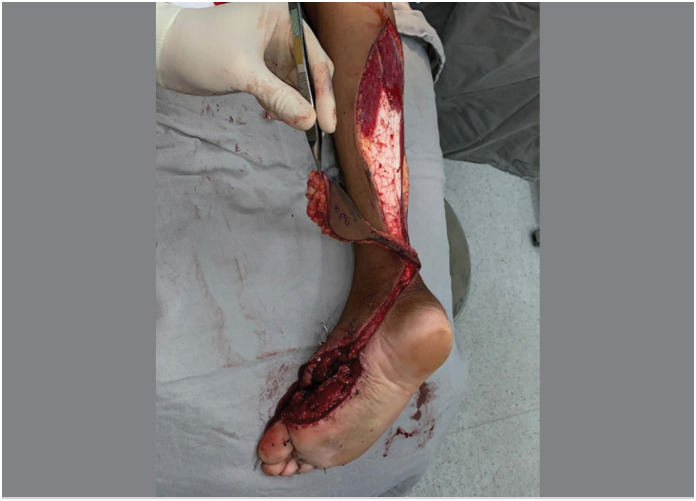
The intact skin between the recipient and donor sites was incised to allow the accommodation of the pedicle.

**Figure 4:**
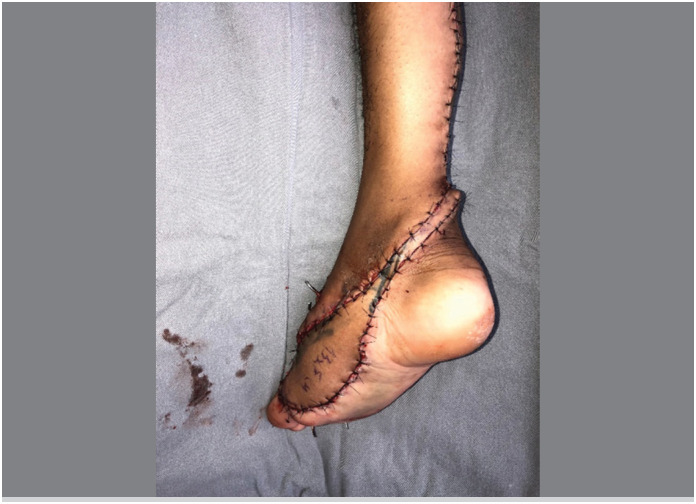
The strip of skin over the pedicle was sutured to the undermined skin between the recipient and donor sites without any tension. The donor site was also primarily closed.

The main aspect of this technical modification was the extension of the flap's skin island over the entire length of the pedicle, as a longitudinal strip of skin in continuity with the flap itself, like a “squash racket “ (Figure 1). The width of this strip of skin was planned to cover both the sural nerve and the lesser saphenous vein, including the vascular network between them (Figure 2). The flap dissection was performed in standard fashion, from proximal to distal, with care to maintain the connection between the strip of skin and the neurovascular pedicle and its connective tissue. After the complete dissection of the flap and its covered pedicle, the flap's transposition was simulated over the intact skin between the recipient and donor sites. The intact skin was then incised over the predicted course and the flap inset was performed (Figure 3). The strip of skin over the pedicle was sutured to the undermined skin between the recipient and donor sites without any tension (Figure 4).

## RESULTS

The average width of the flaps was 4,74 cm, ranging from 9,0 to 3,0 cm, while the average length was 10,22 cm, ranging from 15,0 to 6,0 cm. The average width of the skin strip over the pedicle was 1,61 cm, ranging from 3,0 to 1,0 cm, while the average length was 9,22 cm, ranging from 14 to 5 cm. The donor site of the flaps were primarily closed in all cases. The average follow up was 15 months, ranging 1 to 30 months.

All flaps survived without any major complication or surgical re-intervention. Only one patient presented a minor superficial epidermolysis within the strip of skin over the pedicle, resolved without the need for any debridement or surgical intervention. No skin graft was required to cover the pedicle or donor site of the sural flap in all cases.

The survival rate of the flaps were 100% (20/20). There was no case of total or partial flap loss. The unique case of minor epidermolysis at the pedicle was very limited (1,0 × 0,5 cm) and superficial and resolved spontaneously.

## DISCUSSION

Although very popular and reliable, the traditional sural flap has some shortcomes. The main reported complication is venous congestion resulting in partial or complete flap loss 3,6-8. Therefore, many authors advise against tunnelization of the pedicle during the flap transposition. Instead, they propose a connecting incision between the flap's rotation point and the recipient wound to accommodate the pedicle. In this scenario, variable amounts of skin graft are required to cover the pedicle, since the connecting incision cannot be primarily closed over the pedicle due to the risk of compression.

In 2009, Vendramin^3^ described a technical modification in the sural flap with the inclusion of a strip of skin to cover the flap's pedicle. He observed that the complication rate was reduced from 22.2% to 8.8%.

This study was a prospective cohort of 20 consecutive cases of reverse rural flap using the same technical modification as described by Vendramin and Lee et al 3,9. There was no necessity of tunnelization of the pedicle, since the strip of skin over the pedicle allowed a direct closure of the connecting incision with a tension-free accommodation of the neurovascular bundle. In addition, all patients in this study underwent primary closure of the donor area, requiring no skin graft to cover the vascular pedicle.

There was no flap loss in this study, either complete or partial. The unique case of minor epidermolysis at the pedicle was very limited and superficial. No surgical procedure for flap complication was necessary. If we consider this mild epidermolysis as a complication, we end up with a complication rate of approximately 5%, which is quite lower than previous studies 1,3,6,7. We hypothesize that this strip of skin over the pedicle might be acted as a facilitator for venous drainage as well.

We conclude that this modified sural flap with covered pedicle is feasible and reliable, with a lower rate of complications when compared with the convencional sural flap.
